# Migrant Work Conditions and Health Status—A Longitudinal Study on ‘Dirty Work’ Among Undocumented and Newly Regularized Workers

**DOI:** 10.1007/s12134-024-01182-5

**Published:** 2024-08-10

**Authors:** Mariya Lenko, Jan-Erik Refle, Claudine Burton-Jeangros, Julien Fakhoury, Liala Consoli, Yves Jackson

**Affiliations:** 1 Department of Sociology and Social Research, University of Trento, Trento, Italy; 2 Center for the Interdisciplinary Study of Gerontology and Vulnerability, University of Geneva, Geneva, Switzerland; 3 Swiss Centre of Expertise in Life Course Research, LIVES, Geneva, Switzerland; 4 Department of Sociology, Institute of Sociological Research, University of Geneva, Geneva, Switzerland; 5 Division of Primary Care Medicine, Geneva University Hospital, Geneva, Switzerland

**Keywords:** Health, SRH, Work conditions, Dirty work, Undocumented migrants, Regularization

## Abstract

Migrant workers, particularly undocumented migrants, may be constrained to accept ‘dirty work’. This term refers to poorly paid jobs with long working hours and significant exposure to various risks, potentially affecting their health. While many scholars discuss the health-related risks associated with these work conditions, empirical quantitative evidence is scarce regarding their direct effects on health among undocumented and newly regularized migrants. Consequently, we have formulated the following research question: Does ‘dirty work’ have an influence on the self-rated health of undocumented and newly regularized migrants over time? To answer this question, we utilized a dataset collected during an exceptional regularization procedure in the Swiss Canton of Geneva. Through surveys conducted across four waves, we demonstrate the direct and indirect effects of work conditions on migrants’ health using both random effects and fixed effects models. Our operationalization of ‘dirty work’ reveals that exposure to physically demanding positions has a direct, negative impact on migrants’ self-rated health. This result holds independently of the development of chronic health conditions over time. Our study confirms the existence of a relationship between occupational risks and health and underscores the significance of residence status in this context.

## Introduction

Migrant workers, and especially undocumented migrants, may be constrained to accept jobs that are described as dirty and demeaning due to poor societal status and that include low pay, high work hours, and exposure to occupational risks (Moyce & Schenker, 2018; Rosewarne, 2010). Researchers tend to underscore the occupational risks that are linked to such jobs and their negative influence on migrant health (Ahonen et al., 2009; Sousa et al., 2010; Woodward et al., 2014). However, there is limited quantitative empirical evidence that supports the association between ‘dirty jobs’ and poor migrant health, especially within European contexts. While undocumented migrants report lower health levels than legal migrants or the native population (Kuehne et al., 2015), a link between improved work conditions and better self-rated health (SRH) is not established. Connecting the assumptions about 3D (dirty, dangerous, demeaning) jobs (Ashforth & Kreiner, 1999; Soral et al., 2022) with the concept of ‘dirty work’ coined by Hughes (1951), this paper addresses this missing link and asks whether ‘dirty work’ influences migrant health status over time. Using quantitative panel data collected in line with an extraordinary regularization procedure in the Swiss Canton of Geneva, we can track migrants’ health and their work situation over four waves of panel data, between 2017 and 2022 and over a change in status for parts of the participants. As the population under study consists of undocumented and newly regularized migrants, we assume that these have a high probability of performing dirty jobs due to their unprotected status and as job conditions may not systematically change after regularization.

The paper has three objectives. First, it aims at operationalizing ‘dirty work’ with the help of indicators that are commonly available in surveys. Second, the paper examines the question of how the characteristics of ‘dirty work’ influence migrant health over time. We consequentially use self-rated health as a dependent variable and control for the influence of socio-demographics and prior health status. Third, our article intends to shed light on the relationship between ‘dirty work’ and migrants’ SRH in a European context as most scholarly works concern the USA (Hall & Greenman, 2015; Orrenius & Zavodny, 2009), especially using quantitative datasets. With a number estimated to 15,000, Geneva has a relatively high number of undocumented migrants compared to other Swiss cantons (Morlok et al., 2015), which makes it a particularly interesting case to study. Access to work and hence to economic resources is a primordial element to surviving as an undocumented migrant in this context.

The paper begins with a review of the concept of ‘dirty work’ and its operationalization, followed by an overview of the data used in this article. Our analysis focuses on objective indicators of ‘dirty work’ to elucidate its impact on migrants’ health. These indicators encompass high work hours, low wages, and exposure to occupational risks. Subsequently, these indicators were incorporated into fixed and random effect models to assess their influence on health. Furthermore, we examined whether sociodemographic factors, the status of individuals as undocumented or regularized migrants, and their prior health conditions have an impact on their health status over time.

## Theoretical Framework

### What Is ‘Dirty Work’?

In the literature, different definitions of the concepts of ‘dirty work’ and ‘3D jobs’ exist. Dirty, Dangerous, Demeaning (3D) jobs describe employments that are stigmatized by society and that have low prestige, like sanitation workers (Adepoju, 2003; Ashforth & Kreiner, 1999; Baran et al., 2012; Soral et al., 2022). While 3D jobs describe employment, dirty work focuses on practices that can be inherent to any job. The notion of ‘dirty work’ goes back to Hughes (1951) who introduced and developed the term. He considered that many jobs include at least some ‘dirty’ practices like the incapacity to help others in case of overworked care workers (Baran et al., 2012; Deery et al., 2019; Morriss, 2016). Ashforth and Kreiner (1999), who detail the definition of ‘dirty work’, describe it as work that is linked to physical taint (e.g. contact with death or dangerous substances), social taint (work with stigmatized persons, e.g. prison guard, but equally an activity seen as subordinate, e.g. shoe shiner) or moral taint (work of dubious nature like prostitution or when work is seen as intrusive or offensive, like for bill collectors) (Ashforth & Kreiner, 1999, p.415). Literature developed equally in the direction of what is described as dirty as opposed to meaningful work, as well as towards coping strategies (Laaser & Karlsson, 2021; Soral et al., 2022).

Scholars have shown that dirty jobs are often performed by migrants because of their low attractiveness to the indigenous population (Moyce & Schenker, 2018; Rosewarne, 2010). Or, as Adepoju puts it: “The immigrant workers perform the unglamorous jobs that indigenous people are increasingly reluctant to do” (Adepoju, 2003, p.16). In addition, as residence status often determines access to the formal labour market, undocumented migrants are restrained to informal employment with less protection against abuse (Pelizzari, 2006).

Definitions of what is described as ‘dirty work’ or 3D jobs within a country differ depending on the understanding of what is locally seen as stigmatizing (Berkelaar et al., 2012). Examples from different European countries of low-pay or low-prestige jobs include fruit pickers, retail cashiers, waiters, cleaning jobs, personal care workers, hairdressers, and sewers (Adepoju, 2003; Duemmler & Caprani, 2017; Oesch & Rodríguez Menés, 2011). While those jobs often need low skills, the migrants executing them are not necessarily low-skilled (Adepoju, 2003). Agriculture, food catering, care services, and construction are sectors with high work intensity and are identified as sectors in which many irregular migrants work (Ambrosini, 2010). Dirty jobs all have in common being poorly paid, including excessive work hours and exposure to multiple dangers (Moyce & Schenker, 2018).

The concept of ‘dirty work’ encompasses both a societal perspective, which highlights jobs of low prestige, and an individual dimension. In the individual dimension, specialized workers may find themselves in low-skilled positions that are significantly below their educational qualifications, which they might perceive as demeaning. However, understanding the connection between ‘dirty work’ and the health of undocumented migrants, who are more likely to be employed in such roles, remains limited in European contexts. This limitation is primarily due to the elusive nature of these workers, who employ concealment strategies to avoid detection by authorities (Chauvin & Garcés-Mascareñas, 2014). It is consequentially not surprising that Deery et al. (2019) highlight that most existing studies on ‘dirty work’ are qualitative while quantitative measures of the concept are rare. Even less frequent are longitudinal studies that assess migrant work conditions and health (Drydakis, 2022).

While migrants, and especially undocumented migrants, face exclusion from the formal labour market, it is expected that the options to access different occupations are larger for regularized compared to undocumented workers. Indeed, Erdal and Oeppen (2018) discuss whether 3D jobs are accepted because no other employment options exist and thus represent some kind of coerced occupation, or whether migrants (partly) voluntarily choose to work in 3D jobs. As peers of undocumented migrants often share similar situations, the restriction to specific occupations may be perceived as normal. This relates to the subjective appreciation of whether a job is perceived as demeaning and not valuable, either by society or by individuals. However, taking into account subjective evaluations is difficult since individuals working in such sectors often ascribe a higher value to their jobs than society does and consequently reframe the status of their jobs accordingly (Deery et al., 2019; Morriss, 2016; Soral et al., 2022).

### Operationalization of ‘Dirty Work’

**Fig. 1:**
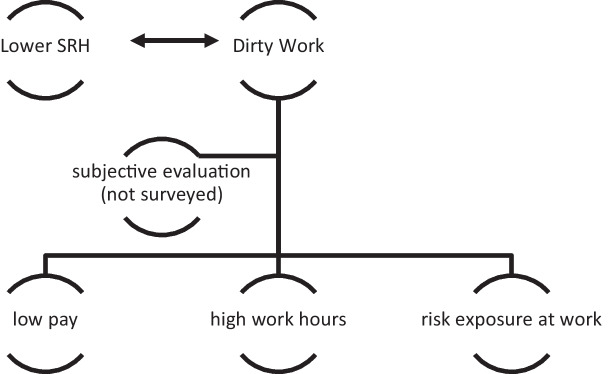
Operationalization of dirty work and link to SRH

The operationalization of the concept of ‘dirty work’ on a subjective level is challenging as we often lack a clear indication of what might be defined as ‘dirty’ and 3D jobs by the society within a country, or even within a region. Similarly, we have no information on whether migrants themselves see their jobs as ‘dirty’ or 3D. For a detailed evaluation of the subjectivity of ‘dirty work’, we would need assessments of both societal and individual evaluations, two aspects where detailed information is lacking. In our study, we also have no evaluation of subjective work across all waves; we therefore turn to measurable objective work conditions. We retain three factors that define ‘dirty work’ as explained by Moyce and Schenker (2018): low pay, high work hours, and exposure to multiple occupational risks. Figure 1 summarizes the operationalization of the concept of ‘dirty work’.

### The Impact of Dirty Work on Migrant Self-Rated Health

Self-rated health (SRH) tends to be particularly lower among undocumented migrant populations compared to the resident population (Jackson et al., 2018; Kuehne et al., 2015). While health levels are found to be higher at the time of arrival in a new country, this tendency diminishes over time (Holz, 2022). Linkages between adverse health outcomes in migrants and arduous work environments have been frequently suggested (Ahonen et al., 2009; Aktas et al., 2022; Sousa et al., 2010; Woodward et al., 2014); however, comprehensive evidence substantiating this connection has remained elusive due to persistent data limitations (Sweileh, 2018; Sterud et al., 2018). Similarly, literature on precarious employment across Europe, but not focused on migrants, found indeed negative effects on health levels (Gunn et al., 2022; Matilla-Santander et al., 2021). Similarly, literature on the health situation of migrants across Europe still faces data scarcity (Lebano et al., 2020). Systematic analyses reveal substantial burdens of psychological and physiological comorbidities alongside elevated injury susceptibilities among migrant workers—findings corroborated through empirical data (Hargreaves et al., 2019; Orenius & Zavodny, 2009).

Historically, scholarship examining migrant worker health initially centred around infectious diseases and healthcare utilization before broadening its focus towards occupational risks (Flynn & Wickramage, 2017). However, the paucity of available data is exacerbated by unique attributes inherent to regional and locally established migrant communities, which exhibit varying socio-demographic profiles hindering attempts at generalizability (Rechel et al., 2011).

Despite these challenges, existent literature suggests that precarious employment situations adversely influence migrants’ health outcomes (Erdal & Oeppen, 2018; Hargreaves et al., 2019; Ornek et al., 2022). Various elements contribute to this negative relationship, including deficient worker protections, temporary statuses, feelings of disempowerment, and imbalanced power dynamics, associated with health outcomes (Ornek et al., 2022). Furthermore, studies reveal that migrant workers encounter heightened vulnerabilities when confronted with chemical exposures at work and diminished decision-making authority compared to native employees—a disparity that predominantly affects male migrants (Ronda et al., 2019). Meanwhile, undocumented female migrants frequently engage in domestic work, entailing them to endure erratic schedules, contact with harsh cleansers, or strenuous physical postures (Hagose et al., 2023).

Research findings suggest links between unfavourable labour settings, mental health issues, and distinct trends shaped by determinants such as profession and gender (Daly et al., 2019; Gkounti et al., 2020). The COVID-19 pandemic exacerbated the pre-existing inequities experienced by migrants, notably affecting their health circumstances (Burton-Jeangros et al., 2020; Kumar et al., 2021). Acknowledging the limits imposed by data accessibility, this paper will concentrate on ‘dirty work’ following the definition established by Moyce and Schenker (2018); regrettably, insufficient information hinders us from addressing broader facets such as power relationships and individual assessments of work configurations.

### Hypotheses

Following the definition of ‘dirty work’, we formulate three hypotheses about its relationships with health. First, we postulate that* very low pay influences self-rated health negatively and once the effective wage increases, self-rated health improves.*

This argument is formulated for several reasons. First, it is expected that a very low wage can be a sign of persistent precariousness that generates economic hardship and a higher prevalence of mental health problems such as depression. This would be even stronger if the pay is perceived as too low for the executed work. Second, the low wage leads to a higher proportion of renouncement of health services due to expected high costs. This is due to the Swiss system of health insurance in which nearly every consultation causes costs to the patient until a certain limit is reached.

Next, we expect that workers with high work hours might suffer from exhaustion and renounce seeking health services due to time constraints, contributing to a worse health condition. Our second hypothesis states that *high work hours negatively influence self-rated health and that a reduction in work hours leads to better self-rated health.*

Last*, we expect that higher exposure to occupational risks leads to lower levels of SRH*. Previous studies have shown that migrant workers frequently face higher exposure to various occupational hazards such as repetitive motions and physical pain (Hall & Greenman, 2015). Continuous exposure to such risks could result in chronic diseases or, in general, worsening of health.

## Material and Methods

### Data

We utilize data collected as part of the Parchemins study, a study designed to assess the consequences of a pilot regularization policy directed at undocumented migrants. This policy was implemented in the Canton of Geneva, Switzerland, between 2017 and 2018 (Jackson et al., 2019). The study gathered data on the health, employment, and social integration of both regularized and undocumented migrants by taking advantage of the natural experiment characteristics of the regularization procedure. This involved having a treatment group (those undergoing regularization) and a control group. The research adopted a mixed-method approach, utilizing both qualitative and quantitative panel data collected over four survey waves spanning from 2017 to 2022. In this paper, we only use quantitative data.

Participation criteria for the study included being a migrant from outside the European Union or European Free Trade Association area, never having sought asylum in Switzerland, maintaining continuous residence in Geneva, and being at least 18 years of age for both regularized and undocumented migrants. It is important to note that not all of the undocumented migrants from the initial survey wave obtained a residence permit over the course of the study, as regularization was contingent on meeting various additional criteria such as demonstrating financial independence, residing in Geneva for a specified duration (10 years, or 5 years for families), and having employment. Switzerland itself has a very large migrant worker population divided between workers with different types of residence permits: short-term permits, temporary residence permits longer than one year and up to several years, border commuters (typically from France in Geneva) and those with a settlement permit that usually lasts 5 years. Through the regularization scheme, migrant workers had the possibility to get a temporary residence permit (‘B-permit’). The regularization scheme targeted the estimated 15,000 undocumented migrants living in the Canton of Geneva (Morlok et al., 2015). In general, workers with foreign nationality in Switzerland account for more than 30% among employed persons (FSO 2024).

Recruitment of participants occurred with the help of migrant organizations that facilitated the application for a residence permit, as well as through a medical centre that provides healthcare services to uninsured migrants free of charge. In general, undocumented migrants do not have access to medical services in Switzerland, except where associations or—as in the Geneva case—a dedicated unit of the cantonal hospital provides medical services free of charge. Former undocumented migrants thus only get access to normal medical services through regularization. Due to the specific characteristics of the targeted participants who fear denunciation and deportation, ensuring trust was essential to recruitment. All participants gave informed written consent to their participation. The data was collected using computer-assisted face-to-face interviews; it was implemented digitally during the pandemic. Interviews were conducted in French, Spanish, Portuguese, or English. The study protocol was approved by the Ethics Committee of Geneva Canton, Switzerland (CCER 2017–00897). A detailed description is published elsewhere (Jackson et al., 2022).

### Sample

For this paper, we make use of quantitative panel data collected over four waves (Wave 1, 2017/2018; Wave 2, 2018/2019; Wave 3, 2020/2021 (between the second and third pandemic waves in Switzerland); Wave 4, 2021/2022). Overall, 468 migrants were surveyed during the first wave of data collection. This number diminished importantly with every wave (Table 1). However, this is not surprising, given the specific profile of this hard-to-reach population of (partly undocumented) migrants that causes high drop-out rates in panel studies (Rothenbühler & Voorpostel, 2016). Reasons for dropout included the lack of time, especially for undocumented migrants compared to regularized migrants, and/or having left the canton of Geneva or Switzerland in between waves. The study however engaged important resources in re-contacting participants several times (for example 6.4 times on average for the second wave) in order to reduce attrition as much as possible (Duvoisin et al., 2023). Due to the evoked reasons, the use of panel data for tracking migrants’ health over time is still limited (Winters et al., 2018), with some notable exceptions in the United States (Bratsberg et al., 2002; Thom, 2010).

**Table 1:** Participation rate by wave

	*N*	%
Wave 1	468	100
Wave 2	375	80.1
Wave 3	309	66.0
Wave 4	259	55.3

Due to the high number of drop-out, we used weighted data for this analysis (for descriptives and statistical models), in order to adjust the results for possible bias arising from longitudinal attrition. The sampling weights were constructed applying techniques of inverse probability weighting (Jenkins & Kerm, 2017; Seaman & White, 2013). Individuals were classified according to their characteristics that could predict attrition, and the retention rate was calculated separately for subgroups of these characteristics. The variables used were study group (control or treatment), sex, level of education, origin, marital status, having a cohabiting minor child in the household, age, length of stay in Geneva, economic precariousness measured by the ability to pay an unexpected bill, self-rated health, and working status. Subsequently, a multivariate analysis was performed in order to test associations between attrition and nominated characteristics. Finally, we constructed weights as a result of the attrition probability (the inverse of the retention probability) at the last wave, predicted by the mentioned characteristics.

**Fig. 2:**
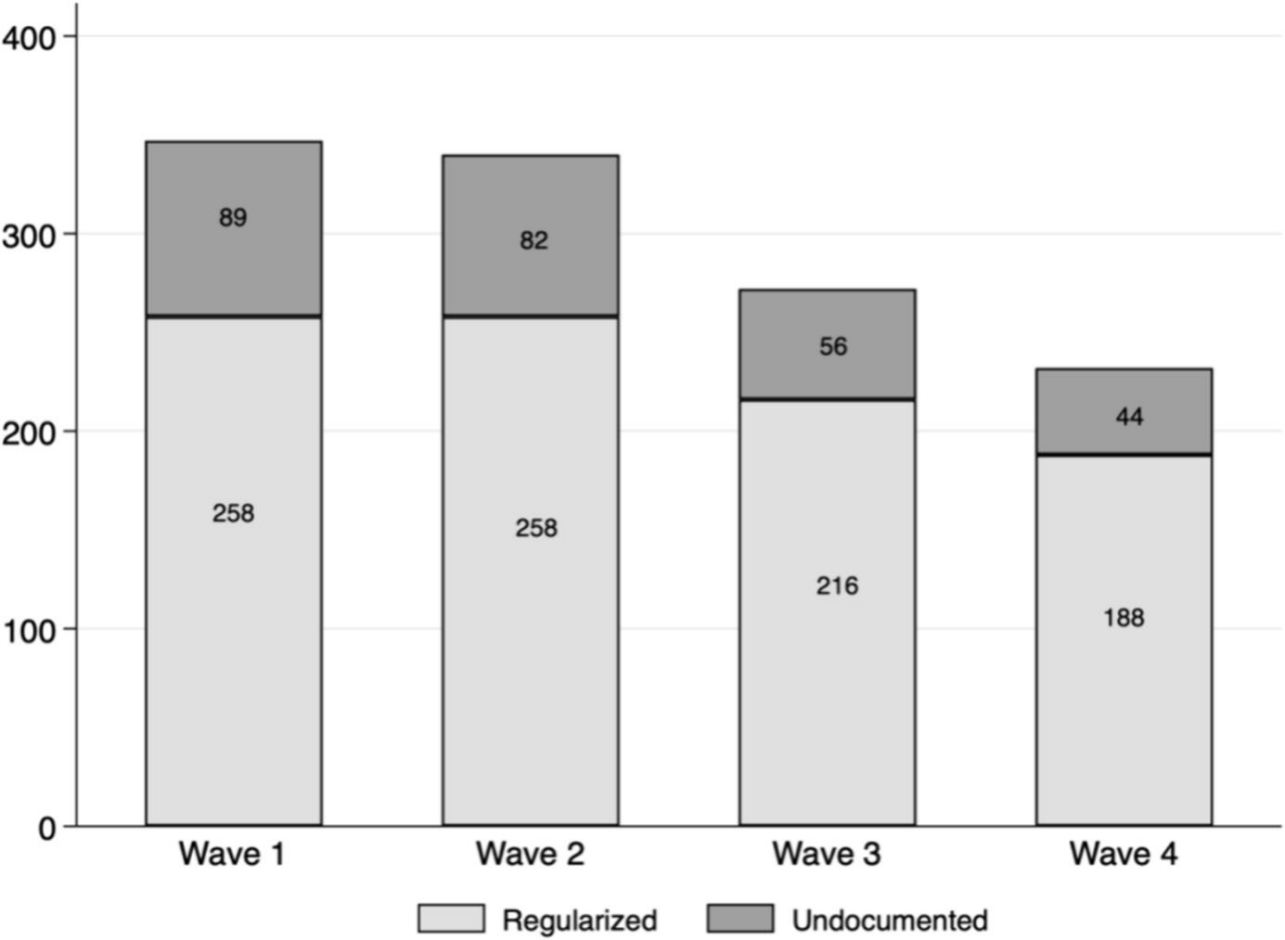
Number of participants by wave and residence status. Regularized includes those that already got a permit or that started the application procedure; undocumented migrants are those that did not apply. Only people having indicated working during at least two waves are included

Since not all migrants were working during all waves, we included only those working during at least two waves in the analytical sample. Migrants who only work occasionally are excluded since the possible impact of regular risk exposure at work remains limited for this sub-group. With this additional criterion, we ended up with a final sample of 368 migrants in our dataset, corresponding to 1191 observations (347 in Wave 1, 340 in Wave 2, 272 in Wave 3, and 232 in Wave 4; Fig. 2). This sample size is rather small but still represents a significant share of the about 3000 people who underwent regularization as part of the regularization scheme. In the following, all descriptives are presented as weighted data.

### Variables

#### Health

In order to shed light on the question of whether ‘dirty work’ influences migrants’ health, our paper focuses on self-rated health (SRH) as the dependent variable. We use the first item from the Short Form Survey (SF-12) with its 5-point scale (Idler & Benyamini, 1997; Sousa et al., 2010) formulated as follows: ‘Overall, do you think your health is…?’ with response items excellent (0), very good (1), good (2), fair (3), and poor (4). We decided to keep the five categories that allow us to assess possible fluctuations across response categories over time (Cullati et al., 2020).

#### Low Pay

By using “low pay”, we aim at identifying workers who have a below-average effective wage. The personal effective wage is the income a regularized or undocumented person gets from paid work. The difference is however that in the case of regularized migrants, wages tend to be higher but include social charges, while in the case of undocumented migrants, wages are lower, but no social charges and taxes are paid. As high work hours are assessed separately, we use an individual monthly wage instead of an hourly wage that would have combined work hours and salary. In terms of low-paid jobs, we use the Genevan poverty threshold, set at 2293 CHF in 2018 (= 1943 EUR, 2008 USD in 2018, BfS, 2020) and create a binary variable.

#### High Work Hours

They are defined as working regularly more than 50 h per week. The Swiss average lies at 43 h/week for full-time employees (BfS, 2022). We set a higher threshold to identify excessive work hours among migrant workers. This workload is found among approx. 10% of workers across waves. While this variable does not assess the intensity or the responsibility overtaken by the worked, we argue that in line with the low-skill sectors in which migrants are working, their workload is more important than their responsibility at work.

#### Occupational Risks

The third aspect of ‘dirty work’ pertains to risk exposure within the workplace. The questions, pulled from the Swiss Health Survey (SHS), encompassed various risks such as noise, exposure to toxic substances, passive smoking, temperature extremes (both high and low), vibrations, heavy lifting, prolonged standing, repetitive movements, and painful or fatiguing positions. The SHS offered the following response options: ‘All the time or almost all the time, about ¾ of the time, about ½ of the time, about ¼ of the time, never or almost never’. To simplify the analysis, we coded the response options into: never, rarely, often, and almost all the time.

The prevalence of these different hazards varies significantly among the participants, with the most common risks being prolonged standing, repetitive movements, exposure to toxic substances, and painful or fatiguing positions (Table 2). Using exploratory factor analysis on the 10 original risk variables (based on their original 4-point Likert scale coding), we identified three distinct groups of occupational risks, each with eigenvalues of 3.2, 1.5, and 1. Further analysis allowed to select two items for each of the three dimensions, leading to the following groupings: exposure to high or low temperatures (risk group 1), heavy lifting and painful or fatiguing positions (risk group 2), and prolonged standing or repetitive movements (risk group 3). The scale reliability coefficients for each risk group are 0.6, 0.7, and 0.7, respectively.

**Table 2:** Exposure to occupational risks (often or always present) across the four study waves

Item	Wave 1%	Wave 2%	Wave 3%	Wave 4%
Noise	3.46	6.38	4.07	4.89
Toxic substances	9.70	22.07	11.90	14.99
Passive smoking	3.21	4.11	2.17	2.58
Low temperature	0.89	1.31	1.81	2.21
High temperature	1.78	4.56	2.58	2.00
Vibrations	3.49	8.47	3.93	5.53
Heavy loads	4.37	10.65	8.56	8.38
Remain standing	68.14	70.38	67.62	43.59
Repetitive movements	55.92	63.87	55.51	40.73
Painful or tiring positions	9.30	13.30	9.89	10.37
*n*	347	340	272	232

We assume these different factors commonly occur in similar working sectors, and the different risk groups may thus correlate with similar health problems. We contend that infrequent risk exposure has no significant impact on health; therefore, we focus on regular exposures. Consequently, we created three dichotomous variables indicating frequent exposure (often or nearly always) to at least one item within each risk group.

#### Controls

In addition to our main variables of interest, we check on differences between regularized and undocumented migrants in order to assess whether residence status matters. It is reasonable to expect that regularized migrants face fewer risks at work, as employers might be keener to respect the labour law. Additional control variables include sex level of education (primary, secondary and tertiary), origin (Eastern Europe, Latin America, and East Asia), and length of stay in Geneva (in years at baseline). In addition, an age group variable, distinguishing younger (18–35), middle-aged (36–50), and relatively older (> 50) workers, is included. In the last step, health-related variables that potentially influence SRH are controlled, including the Body Mass Index (BMI), and multi-morbidity measured as the presence of 3 or more chronic diseases (Fortin et al., 2012). As a measure of mental health, the presence of depression or anxiety, a dichotomous variable, based on self-evaluation was included. Respondents were asked if they were concerned by depression or anxiety or if a health professional had diagnosed them as suffering from these health problems. Health-related variables are included to assess whether changes in evaluations of SRH are persistent when controlling for prior health conditions.

### Statistical Analysis

We use fixed and random effect regression models. The models show intra- and inter-individual changes over time for the included variables. Fixed effect model estimators are a particularly reasonable method in the context of causal inference (Gangl, 2010), notably when observing changes in dependent and independent variables. In the following, we present four models. The first includes risks and work conditions only, the second controls for residence status, and the third adds sociodemographic factors. At last, we include health-related variables to control for the prior health situation. In order to decide which specification is preferable, random or fixed effects, we performed the Hausman test. The Hausman test did not show a clear preference for only one kind of estimator across all models: the results indicated a strong preference for the FE estimator for the first and fourth models and a greater preference for the RE estimator for the second and third models. For better presentation and interpretation, we prefer displaying results for both estimators for each model, allowing a wider opportunity for comparison. Our panel is relatively small, we consequentially work with 90% confidence intervals. All the analyses are run using Stata (version 17). In addition to the fixed and random effect models, we ran several checks to assess the robustness of our models. For instance, we investigate whether the use of the dichotomous dependent variable (being in fair or poor self-rated health) instead of the continuous one could affect findings. The results show that, even if the coefficients are often lower, the main independent variables still remain significant (except in model 4 FE) and maintain the same sign. Similarly, the Hausman test shows the same trends in preferences as described for the continuous variable.

## Results

### Distribution of Main Variables Over Time

Levels of SRH fluctuate only slightly over the four waves, with an average level of about 1.66 (indicating a health status between very good and good) (Table 3). Interestingly, the tendency is not affected by the COVID-19 pandemic in 2020 (Wave 3). As to our independent variables, the share of participants with very low monthly wages fluctuates between 31 and 39% over waves (Table 4). The higher values are found in Waves 1 and 3 (the third wave including COVID-19 pandemic work-related measures). While low pay is much more frequent than high work hours in the sample, the impact of the COVID-19 pandemic is reflected in the lowered share of people with high work hours in Wave 3. We verified whether the excessive work hours are linked to specific sectors. It was indeed the case for the hospitality sector over the first two waves, but that changed radically with the arrival of the pandemic when no surveyed respondent working in this sector indicated excessive work hours in Wave 3. The exposure to occupational risk groups is heterogeneously distributed among our population. Risks in the third group (remaining standing or/and repetitive movements) are reported by around 75% of participants in the first three waves and decreased to around 50% in the last wave. Heavy loads and/or painful positions and exposure to high and/or low temperatures are reported correspondingly by 12% to 18% and around 3–5% of participants across waves.

**Table 3:** Evaluation of self-reported health and dirty work variables across the four waves in the whole study population

	Wave 1 (*N* = 347)	Wave 2 (*N* = 340)	Wave 3 (*N* = 272)	Wave 4 (*N* = 232)
SRH (mean, standard error)	1.68 (0.03)	1.62 (0.03)	1.66 (0.03)	1.64 (0.04)
Monthly wage < 2293 CHF (%)	37.84	36.67	39.03	31.30
Excessive work hours: 50 h + (%)	11.36	11.71	8.62	10.5
Risk group 1 (%)	2.67	5.22	3.21	3.79
Risk group 2 (%)	12.01	18.14	14.75	14.20
Risk group 3 (%)	75.80	79.48	74.48	52.17

**Table 4:** Associations between self-rated health and dirty work variables using random effects (RE) and fixed effects (FE) models

	Model 1		Model 2		Model 3		Model 4	
	RE	FE	RE	FE	RE	FE	RE	FE
Low wage	0.19***	0.10	0.16***	0.10	0.16***	0.10	0.13***	0.09
High work hours	0.08	0.05	0.07	0.05	0.06	0.05	0.07	0.08
Risk group 1	− 0.04	− 0.03	− 0.07	− 0.04	− 0.06	− 0.04	− 0.08	− 0.06
Risk group 2	0.24***	0.21***	0.24***	0.21***	0.23***	0.21***	0.18***	0.17**
Risk group 3	0.04	0.01	0.03	0.00	0.04	− 0.00	0.03	− 0.00
Regularized			− 0.23***	− 0.19*	− 0.23***	− 0.20*	− 0.18***	− 0.20*
Men					− 0.00	(om.)	0.04	(om.)
Education^a^								
Secondary					− 0.02	(om.)	− 0.03	(om.)
Tertiary					0.07		0.03	
Age^b^								
36–50					0.10*	0.11	0.07	0.10
> 50					− 0.01	0.07	− 0.05	0.02
Origin^c^ Latin America Africa Asia					0.36*** 0.45*** 0.23*	(om.)	0.29*** 0.44*** 0.20*	(om.)
Length of stay					− 0.00	(om.)	0.00	(om.)
BMI^d^ Overweight Obese							0.08** 0.16***	0.19** 0.13
Multi-morbidity							0.33***	0.19**
Depression or anxiety							0.34***	0.19**
Constant	1.52***	1.57***	1.73***	1.73***	4.08	1.66***	− 0.88	1.52***
*N* obs *N* groups	1191 368	1191 368	1191 368	1191 368	1191 368	1191 368	1191 368	1191 368

^***^p < 0.01, **p < 0.05, *p < 0.1

*om.* omitted

Reference categories: ^a^primary education, ^b^age group 18–35 y.o., ^c^from Eastern Europe, ^d^normal weight

### The Impact of 'Dirty Work' on Self-Rated Health

Table 4 presents the results of the random (RE) and fixed (FE) effects models examining the connection between ‘dirty work’ conditions and SRH. In a stepwise process, additional variables are introduced into the initial model. The first RE model includes only the primary explanatory variables, i.e., the indicators of ‘dirty’ jobs. Individuals with low wages and those regularly exposed to painful positions report poorer SRH. However, only the results for individuals exposed to the second risk group, involving painful positions or heavy loads, are statistically significant (*β* = 0.24, significant at the 0.01 level).

These coefficients remain significant even when incorporating the variable related to residence status in the second RE model (model 2 RE). The second model reveals that regularized individuals report better health conditions (*β* =  − 0.23, significant at the 0.01 level). Controlling for sociodemographic and health conditions does not alter the levels of significance for low wages and the second risk group. In fact, in the fourth RE model, the estimates are slightly lower but still remain significant.

As expected, respondents who report being overweight, suffering from multi-morbidity, or depression report lower levels of SRH. As mentioned previously, the Hausman test indicated a preference for FE estimators in models 1 and 4, while it favoured RE estimators in models 2 and 3.

In the fixed effect (FE) models, among the primary independent indicators of ‘dirty work’, only exposure to the second risk group has a notable impact on self-rated health. Regular exposure to painful positions or heavy workloads at the workplace has a detrimental effect on SRH (*β* = 0.21, significant at the 0.01 level). This effect remains consistent when accounting for residence status and sociodemographic characteristics (*β* = 0.21, significant at the 0.01 level). In the comprehensive model, which includes health-related variables, this effect slightly diminishes (*β* = 0.17, significant at the 0.05 level). Similarly, as expected, health-related variables such as obesity, multi-morbidity, or depression are associated with a more negative self-evaluation of health. Finally, in all the FE models, the impact of regularization on SRH is significant at the 0.1 level. However, when considering confidence intervals, the intervals include both positive and negative values in this case. The positive extremes range between 0.01 and 0.03 between models, while the negative ones range between − 0.41 and − 0.42. This effect requires further verification in future studies using FE models notwithstanding its persistent effect in RE models.

## Discussion

Our paper aimed to move beyond frequent assumptions about undocumented and precarious migrants working in 3D jobs and the impact of their work conditions on self-rated health. Our first hypothesis, suggesting that low pay influences self-rated health, is partly supported, as individuals with low pay reported lower levels of health in random effect models. However, this effect is not observed in the fixed effect model adopting a longitudinal perspective. Verification with wage as a continuous variable yielded no significant results in the fixed effect model. This finding might point to an inverse relationship where higher health levels influence income levels, particularly among men (Jäckle & Himmler, 2010; Rodriguez-Alvarez & Rodriguez-Gutierrez, 2018). Still, we underscore that the result remains significant when controlling for multi-morbidity in the RE models.

Our second hypothesis focused addressed the significance of high work hours as a component of ‘dirty work’ and its influence on self-rated health, at least as an independent factor. Findings suggest that this effect is not directly observable. Nevertheless, we demonstrate that with a threshold of 50 work hours per week, a range still reported by 8 to 12% of our population, high work hours do not have any direct negative consequences for self-rated health. This finding is surprising given that qualitative work indicated that long work hours are an important problem (Porthé et al., 2010; Harrison & Lloyd, 2012). Still, it could be that the effects of work overload become visible only later in the life course.

Concerning our third hypothesis and more surprisingly, results show that only some forms of risk exposure influence SRH, and this notwithstanding the exposure faced years before participating in the study. Findings show that only regular exposure to painful positions or heavy loads negatively affects SRH among the studied migrant population.

To summarize, low wages and exposure to physically demanding positions and heavy workloads are linked to poorer health, whereas this is not the case for extended work hours and other work-related risk exposures. These findings remain significant even among people developing chronic conditions over time. As anticipated, a deterioration in overall health directly affects individuals’ self-rated health. Although initially included as control variables, chronic health conditions exhibited significant effects in both fixed and random-effect models. Given the established links between chronic health conditions and SRH in the literature (Mavaddat et al., 2014; Perruccio et al., 2012), this is not surprising. These variables were included to test whether risk-related effects have independent effects on individuals’ health evaluation, which is indeed the case for our sample.

Differences between random and fixed effect models provide important insights from a longitudinal standpoint. While the random effect models confirm the importance of work-related risks in our dataset, a significant influence on self-rated health (SRH) can only be established for alterations in employment involving painful positions in the fixed effect models. The connection between painful positions and SRH is common in domestic work, which is a frequent occupation among our study participants. This relation may thus depend on the demographic composition of migrant populations.

Our study presents some limitations. Importantly, we could not measure subjective evaluations of ‘dirty’ work. This pertains to several variables that would be valuable for future studies. These could include assessments of whether individuals perceive their job as valuable, evaluations of the job’s value in society, and inquiries about work autonomy and interpersonal relationships with other workers (Drydakis, 2022). More detailed information on the actual occupation of migrants, as well as information on their initial job choice and priorities for employment, would be useful. We would like to exercise caution by acknowledging that our findings may be influenced by the unique characteristics of the undocumented migrant community in Geneva. Similarly, we used non-random sampling for recruitment (for both migrants undergoing regularization and those having similar characteristics). Therefore, these results may not be easily generalized, as our sample is not representative of all migrant populations. Moreover, a majority of the participants are women employed in the domestic sector, where the nature of risks differs from those in agriculture or industrial occupations, for instance. Additionally, it remains uncertain whether, within the context of Geneva’s exceptional regularization programme, employers have taken measures to reduce risks for undocumented workers to prevent accidents or health issues that could potentially expose the illegality of their work arrangements.

Our results must be read against the rather limited time frame of observation, especially since health problems can manifest over longer periods and might thus become manifest later in the life course, including at the time of retirement. It is likely that participants who have occupied such ‘dirty jobs’ over a long period—considering they have on average been living in Geneva for 12 years—should already be impacted by their consequences. At the same time, poor health conditions could equally have led some to the return to their country of origin. Along with the healthy immigrant effect which assumes that selectivity makes only those in good health migrate to other countries (Kennedy et al., 2015; McDonald & Kennedy, 2004), we could expect that only the healthier ones are able to manage harsh living conditions over a long time frame. Furthermore, other changes happening after regularization may occur with a time lag, e.g. higher salaries or better work conditions. To account for the long-term consequences of difficult job conditions, consistent tracking of migrants over several decades would thus be necessary.

Our findings add information about the health status of newly regularized and still undocumented migrants while considering some characteristics of their jobs in a European context. Furthermore, we show the effects of ‘dirty jobs’ on health, as well as of some other health-related variables using a longitudinal perspective. To our knowledge, such a detailed and longitudinal assessment of changes in health with ‘dirty work’ is still scarce. Besides the objective indicators used here, local assessments of what counts as ‘dirty work’ and for whom are needed (Soral et al., 2022). Furthermore, the self-ascription of being healthy is a precondition for their work, meaning that indicating good SRH could be a self-fulfilling indicator, which is only re-evaluated in case of morbidities.

Finally, our study provides important results for policy formulation. In addition to the known relation between low salaries and health, our findings indicate that exposure to painful positions and heavy loads at work has direct negative consequences, while other risks may be less harmful in a short time frame or in our specific context. It is reasonable to expect that policies targeting exposure to painful positions can prevent negative health consequences for the population in question. Again, we add that a careful analysis of the contexts, that might differ from our case in Geneva, is necessary before applying similar policies.

## Conclusion

This study represents an original research contribution within a European context, focusing on the work-related determinants of health. Initially, we explored the concept of ‘dirty work’ and subsequently delved into how such work may impact self-rated health. Our operationalization of this concept, along with the differentiation of three dimensions (economic precariousness, long working hours, and specific occupational risks), yielded significant empirical insights. Our findings revealed that only specific work-related risks affect the self-rated health of undocumented or newly regularized migrant workers uniformly. Exposure to physically demanding positions and heavy workloads was found to be predictors of poorer self-rated health (SRH). Findings revealed that certain precarious work conditions can indeed have a detrimental effect on self-rated health. They underscore the need for targeted policies aimed at safeguarding migrant workers from low-paying and hazardous employment such as working in physically demanding positions and facing heavy workloads. The results also ask for ergonomic interventions, albeit difficult to realize among undocumented migrants.

## Data Availability

An anonymized version of the dataset is available under: Jackson, Y.-L. J., Burton-Jeangros, C., Duvoisin, A., Refle, J.-E., Consoli, L., & Fakhoury, J. (2024). Parchemins study: impact of legal status change on undocumented migrants’ health and well-being (Version 1.0.0) [Data set]. FORS data service.
